# Obesity, rather than high fat diet, exacerbates the outcome of influenza virus infection in influenza-sensitized mice

**DOI:** 10.3389/fnut.2022.1018831

**Published:** 2022-10-20

**Authors:** Weimin Guo, Dayong Wu, Lijun Li, Samuel Ding, Simin Nikbin Meydani

**Affiliations:** ^1^Nutritional Immunology Laboratory, JM USDA Human Nutrition Research Center on Aging, Tufts University, Boston, MA, United States; ^2^Department of Medicine, Tufts University School of Medicine, Boston, MA, United States

**Keywords:** high fat diet, obesity, influenza virus, infection, vaccination, animal model

## Abstract

**Introduction:**

Obesity is associated with impaired immune function and increased susceptibility to infection. High fat (HF) diet-induced obesity is a commonly used animal model. However, HF diet itself is known to affect immune function and infection. Thus, it is not discernable which one, HF diet or adiposity, is the major contributor to the observed impairment in immunity and susceptibility to infection in HF diet-induced obesity. We hypothesized that obesity is a major contributor to impaired immune function.

**Methods and results:**

Weight-matched outbred female CD-1 mice (1-mo) were randomly assigned to either a HF (45%) or a low fat (LF, 10%) diet group. Ten week after feeding their respective diets, weight gain in the mice fed the HF diet varied greatly. Thus, based on the average body weight, mice in HF diet group were divided into two sub-groups: HF lean (HF-L) and HF obese (HF-O). After 25-week, mice were immunized with an influenza A/Puerto Rico/8/34 vaccine and boosted 3-week later. Five week after the booster, mice were infected with influenza A/Puerto Rico/8/34 virus, and body weight was recorded daily for 1 month. HF-O mice exhibited significant weight loss after influenza virus challenge compared to LF and HF-L mice while LF and HF-L mice largely maintained their weight to a similar extent.

**Conclusion:**

Our findings suggest that obesity, rather than HF diet, *per se*, may impair the efficacy of influenza vaccination.

## Introduction

Accumulating evidence from animal and human studies suggests that obesity is associated with impaired immune function and increased susceptibility to infection (1–6). Diet-induced obese mice exhibit increased mortality and altered immunity in response to influenza infection (7). Vaccination is the most efficient means for protection against influenza infection, vaccinated obese mice, however, are less protected from influenza virus challenge and have delayed viral clearance compared to lean mice (8, 9). Decreased influenza vaccine efficacy in obese people may be partially due to reduced activation, maintenance, and activity of memory T cells (10, 11). Mice are commonly used as an animal model for influenza infection. Weight loss and subsequent recovery are key clinical indicators for monitoring the severity and progression of influenza-infection in mice (12–15). Recently, measurement of cell mediated immunity by quantitating *in vitro* cytokine production such as IFN-γ has been used to assess vaccine efficacy (16, 17).

The high-fat diet-induced obesity rodent model is the most commonly utilized animal model for the study of obesity-related disorders, which involves feeding mice a high fat (HF) diet (typically providing 45 or 60% calories from fat). However, high fat by itself is also known to affect immune cell function (18–20). For example, HF diet feeding increased the expression of pro-inflammatory cytokine such as tumor necrosis factor-α (TNF-α) and interferon-γ (IFN-γ) (21). Therefore, it is difficult to determine whether, and to what extent, the reported adverse impact of obesity on influenza infection is due to influence of obesity on immune function and resistance to infection, or a direct result of the high fat content of the diets used, or both.

We had an opportunity to investigate this question as part of a HF-induced obesity animal study using CD-1 mice. We noted that these outbred mice had highly heterogeneous weight gain in response to HF feeding. Briefly, female CD-1 mice (1-month-old) were weight-matched and fed a HF (45% kcal fat) or low fat (LF, 10% kcal fat) control diet. After 10-week of feeding, the CD-1 mice fed the HF diet had considerable variation in the degree of weight gain. Based on the average body weight, we further divided the obesity-prone and obesity-resistant CD1 mice fed the HF diet into two sub-groups: HF obese (HF-O) and HF lean (HF-L). Utilizing this mouse model we investigated the effect of obesity and high fat on immune function and response to viral infection. We hypothesized that obesity is a major contributor to the impaired protection against influenza challenge. Our findings indicate that obesity, rather than HF diet, *per se*, may impair the efficacy of influenza vaccination.

## Methods

### Animals and diets

**FIGURE 1:**
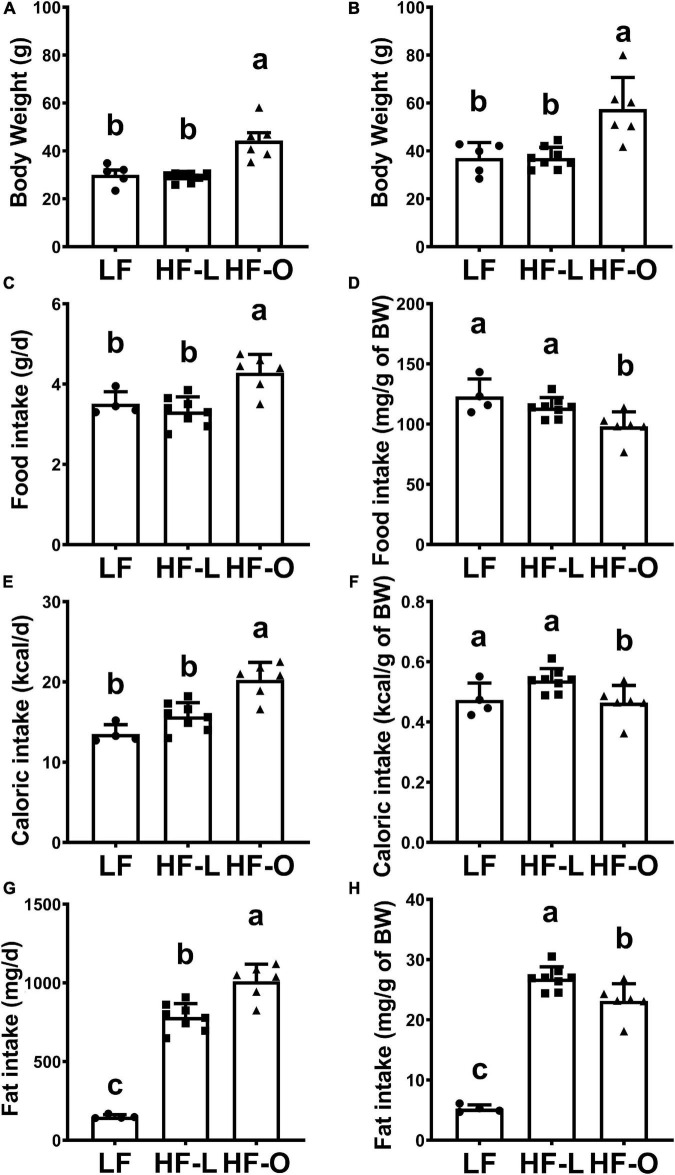
High fat diet-induced obesity in CD-1 mice. Body weight of mice fed LF and HF diets for 10-week **(A)** and for 25-week **(B)**. Food intake **(C,D)**, caloric intake **(E,F)**, and fat intake **(G,H)** of mice were measured after 10 weeks of feeding the diets. Values are means ± SD, *n* = 4–8. Labeled means without a common letter differ at *P* < 0.05.

Four-week-old outbred female CD-1 mice (17∼22 g, *n* = 20) were purchased from Charles River Laboratories. Mice were individually caged and had free access to diet and water. All experimental procedures involving animals were approved by the Animal Care and Use Committee of Tufts University and conducted according to the NIH Guidelines for the Care and Use of Laboratory Animals (approval number H2013-135). Mice were randomly assigned to two groups: Low fat (LF, 10% calories from fat, Research Diets, #D12450J) diet (*n* = 6) and high fat (HF, 45% calories from fat, Research Diets, #D12451) diet (*n* = 14) group. The compositions of the two purified diets are provided in Supplementary Table 1. During the feeding period, body weight was recorded weekly. After 10-week of feeding with their respective diets, body weight increased in all mice but the weight gain in HF group varied greatly. Based on the average body weight of the obese mice, mice in HF group were further divided into HF lean (HF-L, BW < 34.6 g) and HF obese group (HF-O, BW > 34.6 g) (Figure 1A).

Next, daily food intake was recorded for three consecutive days. Total food intake (g/day), relative food intake (g/g of body weight per day), and total caloric intake (kcal/day), and relative caloric intake (kcal/g of body weight per day) were determined. Mice were fed their respective diets for another 15-week, at which time they were immunized twice with influenza vaccine and infected with influenza virus as detailed below.

### Vaccination and viral infection

After a total of 25-week of feeding, mice were immunized with an influenza A/Puerto Rico/8/34 vaccine as previously reported (22). Briefly, the mice were intraperitoneally injected with 200 μL of the influenza vaccine, which was prepared by mixing the hemagglutinin protein of influenza A strain A/Puerto Rico/8/1934 (H1N1) (Sino Biological) and aluminum adjuvant (Thermo Fisher Scientific). The immunization was repeated 3-week later (booster). Blood was collected 4-week after the booster for antibody titer analysis. Five week after booster injection, under anesthesia, mice were infected intranasally with 50 hemagglutinin units of influenza A/Puerto Rico/8/34 virus diluted in 0.05 mL PBS (kindly provided by Dr. Melinda Beck of UNC Gillings School of Global Public Health). Five unvaccinated CD-1 mice fed LF diet were infected with influenza virus as the control group to validate success of infection. Body weight was recorded daily for one month as indicator of severity of infection, and then the mice were euthanized with CO_2_ asphyxiation followed by exsanguination.

### Assessment of serum antibody titer by hemagglutination inhibition assay

Blood was collected after influenza vaccination boost and 30 days post-infection at animal sacrifice, respectively, and the serum was separated and stored in −80°C for determining serum antibody titer. The serum antibody titer was assessed according to published methods (23, 24), with minor modification. Briefly, serum was incubated with receptor destroying enzyme (RDE) (Denka Seiken, Tokyo, Japan) overnight at 37°C to remove non-specific inhibitors of hemagglutination (HA), The remaining RDE enzyme was heat inactivated at 56°C for 40 min, followed by adding PBS for a final 1:10 dilution of the serum. Twenty-five μL RDE-treated serum was twofold serially diluted in v-bottom microtiter plates, and then, 25μL of influenza virus (4 HAU) was added to each well followed by incubation at room temperature for 40 min. Next, 50 μL 1% Turkey red blood cells (TRBC) (Lampire Biologicals, Pipersville) in PBS was added to each well, and the plates were mixed by agitation, covered, and the RBCs were allowed to settle for 45 min at room temperature. HAI titer was determined by the reciprocal dilution of the last row which contained non-agglutinated RBC. Positive and negative serum controls were included for each plate.

### *Ex vivo* cytokine production

Splenic lymphocytes were isolated for assessment of *ex vivo* cytokine production as previously reported (25). Levels of selected T cell cytokines in the cell culture supernatant of the cultured splenic lymphocytes stimulated by LPS, T cell mitogen concanavalin A (Con A), or anti-CD3/CD28 T cell receptor antibodies were determined by ELISA. Briefly, splenic lymphocytes were cultured in the presence of LPS (1.0 μg/mL) for 24 h for inflammatory interleukin (IL)-1β, IL-6, TNF-α production or in the presence of Con A (1.5 μg/mL) or anti-CD3 (5 μg/mL)/anti-CD28 (2 μg/mL) for 48 h for IFN-γ, IL-2, IL-4, and IL-10 production. Cell-free supernatants were collected at the end of incubation and analyzed using ELISA kits (R&D Systems, Minneapolis, MN) for all the cytokines according to the manufacturer’s instructions.

### Statistical analysis

All results were tested for normality and homogeneity of variance. A two-tailed *t* test was used to compare two group means, while the one-way analysis of variance (ANOVA) and Tukey’s *post hoc* tests were performed to compare three group means. Correlation coefficients were calculated by using the non-parametric Spearman correlation. Statistical analysis was performed using IBM SPSS Statistics (version 25) software. Results are means ± SD. Significance was set at *p* < 0.05.

## Results

### CD-1 mice model of high fat diet-induced obesity

After 10-week of feeding the respective diets, mice fed the LF diet weighed 30.1 ± 3.6 g (*n* = 5, one mice fed the LF diet died of unknown cause), whereas those fed the HF diet exhibited high variation in weight gain (34.6 ± 8.2 g, *n* = 14) (Supplementary Figure 1A). Thus, based on the average body weight of the CD1 mice fed HF diet, we further divided the HF-fed mice into two sub-groups: HF lean (HF-L, BW < 34.6 g, *n* = 8) and HF obese group (HF-O, BW > 34.6 g, *n* = 6). The average body weight of HF-L group was similar to that of LF mice, while HF-O mice weighed significantly more than LF or HF-L mice (both at *p* < 0.001) (Figure 1A and Supplementary Figure 1B). Similarly, total food intake (g/day) (Figure 1C) and total calorie intake (kcal/day) (Figure 1E) in HF-O group were significantly higher than those in LF mice and HF-L mice. However, after normalizing to body weight, relative food intake (g/g BW) (Figure 1D) and relative caloric intake (Kcal/g BW) (Figure 1F) of HF-O mice were significantly lower than LF mice and HF-L mice, suggesting that HF-O mice might have less energy expenditure and/or higher energy absorption efficiency. In addition, both HF-L mice and HF-O mice had significantly higher total fat intake (mg/day) (Figure 1G) and relative fat intake (mg/g of BW) (Figure 1H) than LF mice. However, when corrected by body weight, HF-O mice had significantly lower relative fat intake than HF-L mice (Figure 1H). Taken together, these observations suggest that the observed differences in weight between HF-O and HF-L is independent of fat intake.

After the mice were fed their respective diets for another 15-week, the body weight of HF-O mice was still significantly higher than that of LF and HF-L mice while there were no significant differences in body weight between LF and HF-L mice (Figure 1B).

### Vaccinated high fat obese mice exhibited more severe symptom after influenza infection compared to their counterparts in high fat lean and low fat groups

After total of 25-week of feeding, mice were vaccinated with influenza virus and boosted 3-week later (28-week of feeding). Five week after vaccination (33-week after feeding), mice in LF, HF-L and HF-O groups were infected with influenza virus, and a group (*n* = 5) of unvaccinated CD-1 mice fed the LF diet was also infected in parallel to serve as the positive control for infection. The unvaccinated control mice had significantly higher weight loss (a primary symptom of influenza infection in rodent models) than the vaccinated mice after infection. The weight loss started on day 6 post viral infection and continued to day 12 post-infection (peak days), which confirmed the effectiveness of both infection and protection by the vaccine (Supplementary Figure 2). It is worth noting that, although the unvaccinated mice gradually recovered to their initial weight by day 12 post-infection, the mean weight gain of unvaccinated mice before termination of infection study was generally lower than that of vaccinated mice.

**FIGURE 2:**
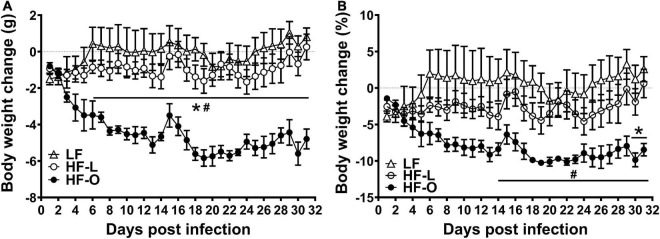
HF-O mice is less protected by influenza vaccine following influenza virus infection. Vaccinated mice fed LF or HF diets were infected with influenza virus 5 weeks after vaccination. Absolute weight loss **(A)** and percent weight loss **(B)** were determined, respectively. Values are means ± SD, *n* = 4–7. ^#^*P* < 0.05 compared to LF mice, *P < 0.05 compared to HF-L mice.

Of the three vaccinated groups, HF-O mice had significantly more weight loss after influenza virus challenge compared to LF and HF-L mice, and there was no significant difference in body weight loss between LF and HF-L mice (Figure 2). Further, Spearman correlation analysis demonstrated significant associations between initial (pre-infection) body weight and infection-induced absolute body weight loss (rho = −0.6321, *p* = 0.01) and percentage weight loss (rho = −0.6143, *p* = 0.01), indicating that the mice with larger initial body weight had more weight loss after infection. These results suggest that obesity rather than high fat content of the diet contributes to more severe infection outcome following vaccination.

### High fat obese mice had significantly higher *ex vivo* interferon-γ production

**FIGURE 3:**
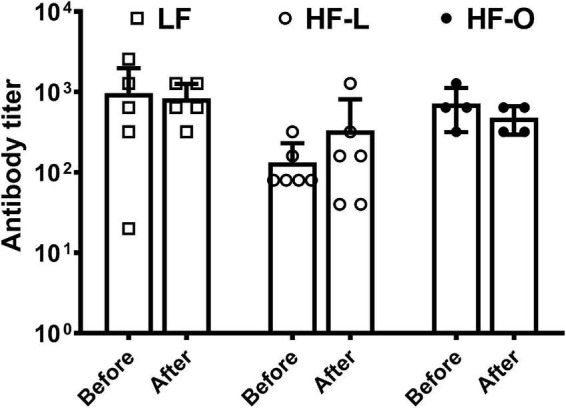
Serum antibody titer by HAI assay. The mice were immunized with influenza A/Puerto Rico/8/34 vaccine. HAI serum antibody titers were determined in the blood collected before viral infection and at the time mice were terminated, referred to as after in the graph. Values are means ± SD, *n* = 4–6.

**FIGURE 4:**
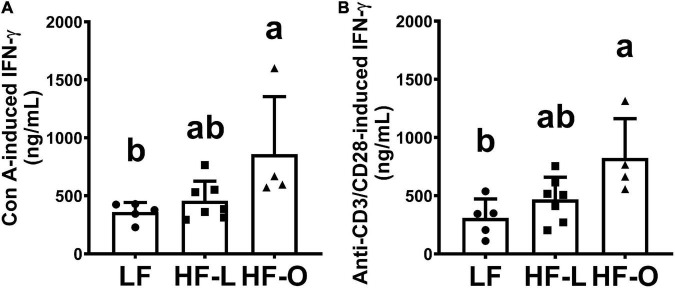
*Ex vivo* production of IFN-γ by splenic lymphocytes. Splenocytes were stimulated by T cell mitogen Con A **(A)** or anti-CD3/CD28 antibodies **(B)**. *Ex vivo* IFN-γ production was determined after 48 h incubation by ELISA, results are expressed as means ± SD, *n* = 4–7. Means with different letters are significantly different at *p* < 0.05.

In order to elucidate mechanism(s) of more weight loss in HF-O mice, we examined antibody titer levels and *ex vivo* T cell cytokine production by splenic lymphocytes. There was no significant difference in antibody titer within the group and among the groups (Figure 3). HF-O mice had significantly higher IFN-γ production in both Con A and anti-CD3/CD28-stimulated splenic lymphocytes compared to LF mice. There was no significant difference in IFN-γ production from Con A or anti-CD3/CD28-stimulated splenic lymphocytes between LF mice and HF-L mice (Figures 4A,B). Further IFN-γ production tended to be higher in HFO mice compared to HF-L mice (*p* = 0.0774 for Con A and *p* = 0.0662 for anti-CD3/CD28 stimulation). In addition, we found that CD3/CD28-stimulated IFN-γ production from splenic lymphocytes was positively associated with final body weight (rho = 0.6536, *p* = 0.01), negatively correlated with absolute body weight loss (rho = -0.5571, *p* = 0.03), and percent body weight loss (rho = −0.5179, *p* = 0.05). These results suggest that obese mice may produce more IFN-γ, which, in turn, might contribute to an inflammatory state and higher body weight loss during influenza infection.

Excepting that HF-O mice tended to have higher TNFα production (215.5 ± 58.9 pg/mL) from Con A-stimulated splenic lymphocytes (*p* = 0.09) compared to LF mice (199.7 ± 42.1 pg/mL), no significant difference was found in *ex vivo* production of other cytokines (IL2, IL-4, and TNFα) among the three groups (Supplementary Figure 3).

## Discussion

Our results suggest that obesity induced by high fat diet, rather than the high content of fat in the diet, may be the main contributor to reduced efficacy of influenza vaccination in mice. Further, our results suggest that this adverse effect of obesity may be mediated through changes in IFN-γ levels.

Studies have shown that obesity is associated with impaired immune response to influenza infection (2, 3, 26), and also decreases efficacy of influenza vaccination as demonstrated in both humans and mouse models (4, 5). While serum anti-influenza antibody has been a key clinical indicator for evaluating vaccine-induced protection, recent findings suggest that serum HAI antibody titer might not always predict protective efficacy of vaccination (27–29). Some other components of the immunity, such as T cell-mediated anti-viral response, may also be responsible for vaccine-induced protection. This may explain our findings that HF-O mice were less protected by vaccination, although there were no significant differences in serum HAI antibody titer compared to LF and HF-L groups.

Unlike Type I IFNs, IFN-α, and IFN-β, two major antiviral cytokines activated during influenza virus infection (30, 31), the Type II IFN, IFN-γ, has a 10–100-fold lower specific antiviral activity than Type I IFNs (32), and it has even been suggested to increase susceptibility to influenza A infection (33). In addition, IFN-γ plays an important role in acute lung injury induced by influenza virus infection, and neutralization of IFN-γ with monoclonal antibodies alleviated acute lung injury in mice (34). Thus, the observed higher *ex vivo* IFN-γ production by splenic lymphocytes of HF-O mice may be related to more weight loss, a key clinical indicator of influenza infection, in these mice.

The results from our current study suggest that obesity, rather than high content of fat in the diet, may contribute to severity of influenza infection in diet-induced obesity mice. Elevated *ex vivo* IFN-γ production might be a contributing factor to severity of clinical symptom of influenza infection, key among which is weight loss, the primary indicator of morbidity and disease severity from infection in animal models of influenza (35–37). This is supported by higher IFN-γ production in HF-O mice compared to HF-L and LF groups as well as significant correlation between IFN-γ level and weight loss observed in this study. On the other hand, we did not observe a significant difference in weight loss and IFN-γ production between mice in HF-L and LF groups. We did not detect significant differences in production of other cytokines among the three groups, suggesting that changes in IFN-γ levels may be the main contributor to the exaggerated weight loss in the HF-O mice. While we observed a trend toward higher TNFα levels in HF-O mice compared to HF-L and LF groups, this difference did not reach statistical significance and no correlation was observed between weight loss and TNFα levels.

We noticed very different variation in body weight gain between obesity-prone (HF-O) mice and obesity-resistant (HF-L) CD1 mice, i.e., HF-O group had much larger variability in the body weights than HF-L group. The mechanism behind this phenomenon is not clear. Previous report showed that there were substantial differences in transcriptomes, phenotypes and metabolic processes between obesity susceptible and resistance C57BL/6J mice fed high fat diet (38). These observations may provide a roadmap for further investigation.

This study was originally designed to determine how maternal obesity impacts offspring’s immune function; however, the greatly varied weight gain in these dams in response to HF diet prompted us to take the opportunity and investigate relative contribution of obesity vs. HF diet to the impaired immune function and increased susceptibility to influenza infection that is associated with diet-induced obesity. It is worth noting that, in addition to one LF mouse that died due to unknown causes before vaccination and infection, we lost one HF-L mouse and two HF-O mice during the vaccination and post infection period. As a result, a limitation of the current study is the relatively small sample size, which may not be adequately powered to detect statistically significant difference for some of the biomarkers tested. Second, our study did not determine the metabolic parameters associated with observed differences in weight loss due to influenza infection, which would have helped determine if there is also a difference in metabolic health between HF-L and HF-O mice. Despite these limitations, the findings of this pilot study provide useful information regarding the impact of obesity rather than high fat content of the diet on obesity-induced impaired immune function and higher susceptibility to influenza infection.

## Data availability statement

The original contributions presented in this study are included in the article/Supplementary material, further inquiries can be directed to the corresponding author.

## Ethics statement

The animal study was reviewed and approved by the Animal Care and Use Committee of Tufts University.

## Author contributions

WG designed the study, performed the experiments, analyzed and interpreted the data, and prepared the manuscript. DW, LL, and SD performed the experiments. DW and SM designed the study, interpreted the data, and revised the manuscript. All authors approved the manuscript as in the submitted version.
