# Supplementary material for: Obesity, rather than high fat diet, exacerbates the outcome of influenza virus infection in influenza-sensitized mice
Source: Front Nutr. 2022 Oct 20;9:1018831. doi: 10.3389/fnut.2022.1018831 (PMC9631825; doi:10.3389/fnut.2022.1018831)
Supplement: Supplementary file 1 [file Data_Sheet_1.PDF]

Supplementary Material

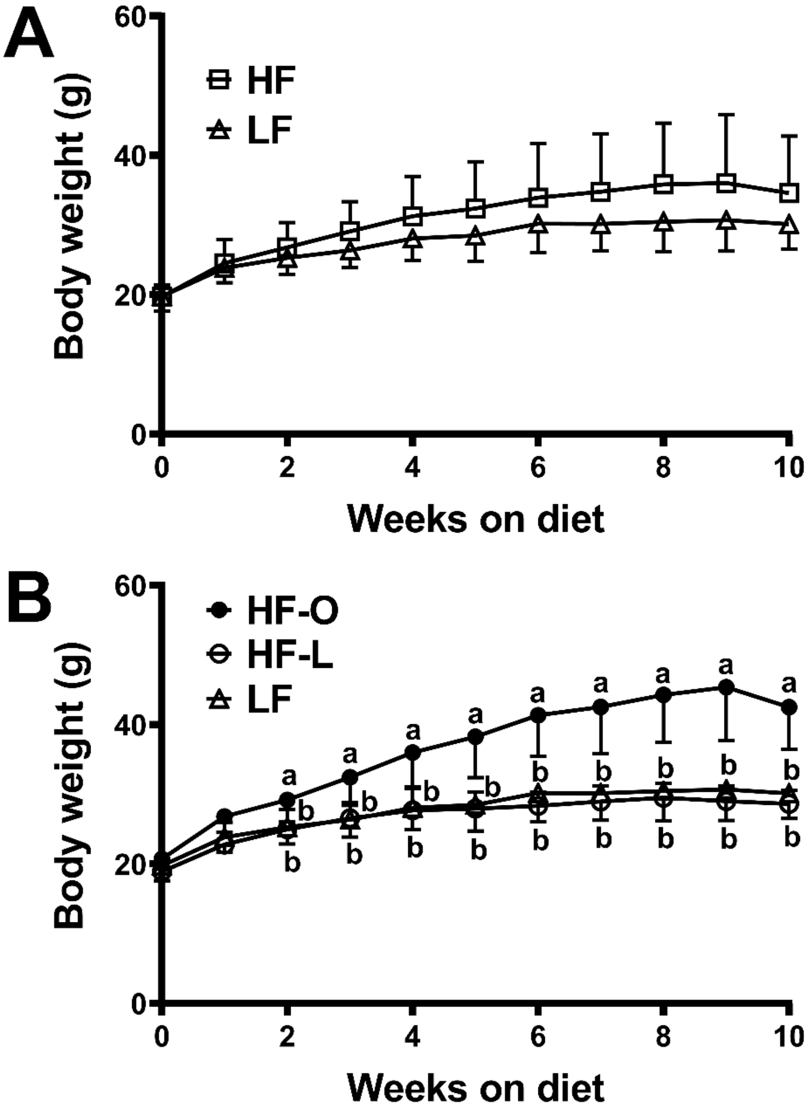

Supplementary Figure 1. Body weight curve.

The CD1 mice were fed LF (n = 5) or HF (n = 14) diets for 10 wks. Mice fed the HF diet exhibited high variation in weight gain (34.6 ± 8.2 g) compared to those fed LF diet (30.1 ± 3.6 g) (A). Based on the average body weight of the mice fed the HF diet, the HF-fed mice were divided into HF lean (HF-L, BW < 34.6 g, n = 8) and HF obese group (HF-O, BW > 34.6 g, n = 6) (B). Values are means ± SD, n = 5 – 8. Means with different letters are significantly different at $p < 0.05$.

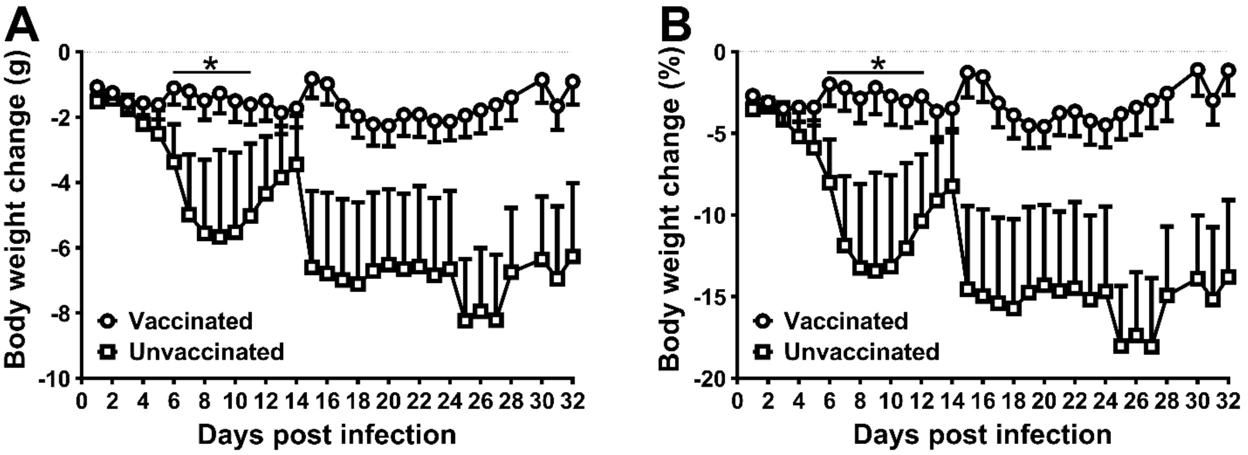

Supplementary Figure 2. Influenza vaccine prevents weight loss in CD-1 mice infected with influenza virus.

Vaccinated mice fed LF or HF diets were infected with influenza virus 5 wks after vaccination. A group of mice fed the LF that were not vaccinated were also exposed to influenza virus. Absolute weight loss (A) and percent weight loss (B) were determined. Values are mean ± SD, n = 4 – 7. * P<0.05 compared to unvaccinated mice.

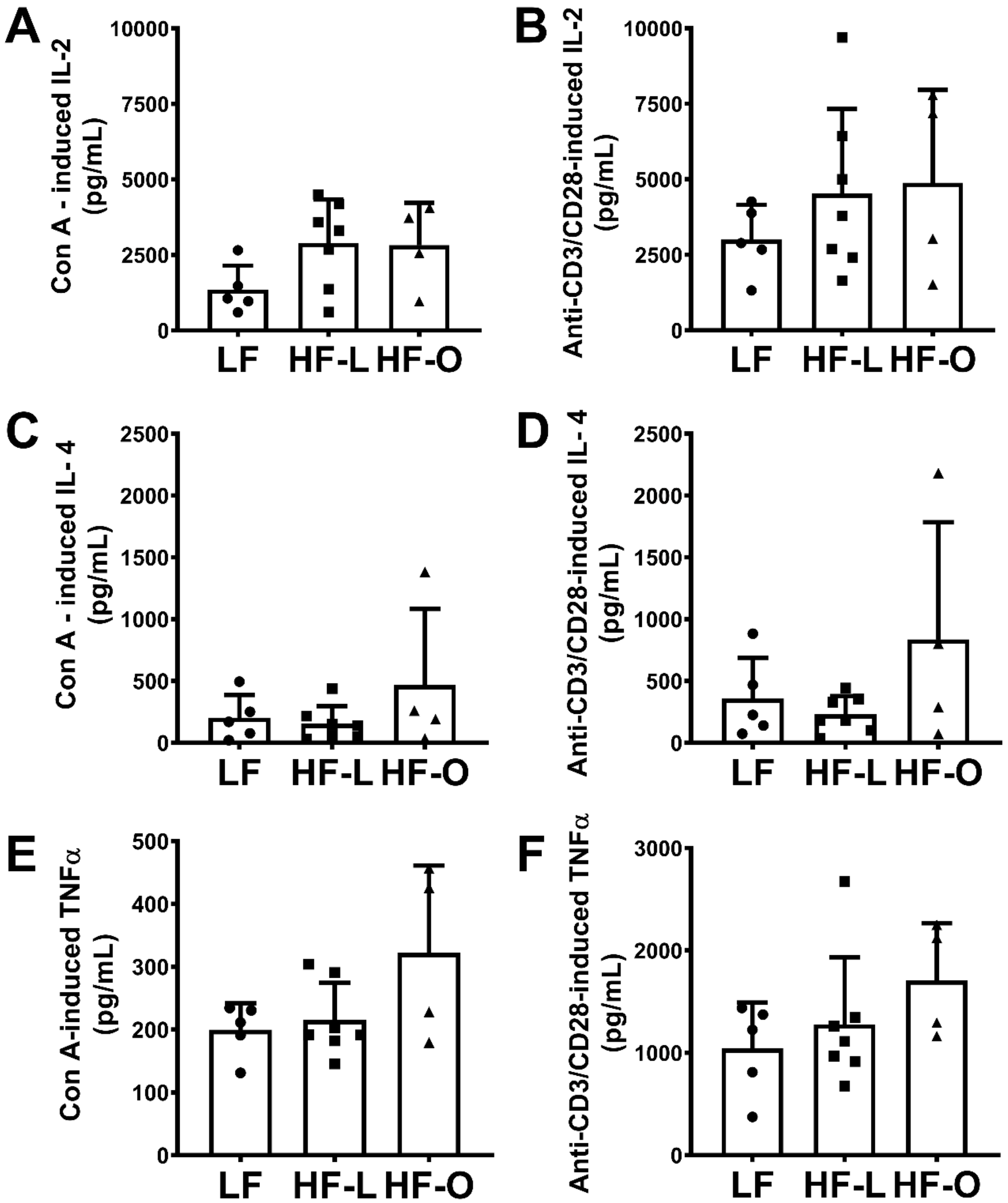

Supplementary Figure 3. Effect of obesity and high fat diet on *ex vivo* cytokine production.

Splenocytes were stimulated by T cell mitogen Con A (A, C, E) or anti-CD3/CD28 antibodies (B, D, F). *Ex vivo* cytokine production was determined after 48 h incubation by ELISA. Values are means ± SD, n = 4 – 7.

Supplementary Table 1   Ingredient composition of experimental diets (g/kg)

Ingredient	LF diet	HF diet
Casein, 30 Mesh	189.6	233.1
L-Cystine	2.8	3.5
Corn Starch	479.8	84.8
Maltodextrin 10	118.5	116.5
Sucrose	65.2	201.4
Cellulose, BW200	47.4	58.3
Soybean Oil	23.7	29.1
Lard	19.0	206.8
Mineral Mix S10026	9.5	11.7
DiCalcium Phosphate	12.3	15.1
Calcium Carbonate	5.2	6.4
Potassium Citrate, 1 H2O	15.6	19.2
Vitamin Mix V10001	9.5	11.7
Choline Bitartrate	1.9	2.3
FD&C Yellow Dye #5	0.038	0.000
FD&C Red Dye #40	0.000	0.058
FD&C Blue Dye #1	0.009	0.000
Total (g/kg)	1000	1000
Total energy (kcal/g)	3.85	4.73

LF, low fat diet (Research Diets #D12450J with 10% energy from fat);

HF, high fat diet (Research Diets #D12451 with 45% energy from fat)
